# Hypertension in an Emergency Department Population in Moshi, Tanzania; A Qualitative Study of Barriers to Hypertension Control

**DOI:** 10.1371/journal.pone.0279377

**Published:** 2023-01-06

**Authors:** Sophie W. Galson, Msafiri Pesambili, Joao Ricardo Nickenig Vissoci, Preeti Manavalan, Julian T. Hertz, Gloria Temu, Catherine A. Staton, John W. Stanifer

**Affiliations:** 1 Duke Emergency Medicine, Duke Global Health Institute, Durham, NC, United States of America; 2 Kilimanjaro Christian Medical Center, Kilimanjaro, Tanzania; 3 Division of Infectious Diseases and Global Medicine, Department of Medicine, University of Florida, Gainesville, FL, United States of America; 4 Duke University Medical Center, Duke Global Health Institute, Durham, NC, United States of America; 5 Munson Nephrology, Traverse City, MI, United States of America; Universita degli Studi di Milano, ITALY

## Abstract

**Background:**

Sub-Saharan Africa has a high prevalence of hypertension with a low rate of awareness, treatment adherence, and control. The emergency department (ED) may represent a unique opportunity to improve hypertension screening, awareness, and linkage to care. We conducted a qualitative study among hypertensive patients presenting to the ED and their healthcare providers to determine barriers to hypertension care and control.

**Methods:**

In northern Tanzania, between November and December 2017, we conducted three focus group discussions among patients with hypertension presenting to the emergency department and three in-depth interviews among emergency department physicians. In our study, hypertension was defined as a single blood pressure of ≥160/100 mm Hg or a two-time average of ≥140/90 mm Hg. Barriers to care were identified by thematic analysis applying an inductive approach within the framework method.

**Results:**

We enrolled 24 total patients into three focus groups and performed three in-depth interviews with individual providers. Thematic analysis identified two major domains: 1) patient knowledge, attitudes, and practices, and 2) structural barriers to hypertension care. Four major themes emerged within the knowledge, attitudes, and practices domain, including disease chronicity, provider communication, family support, and fear-based attitudes. Within the structural domain, several themes emerged that identified barriers that impeded hypertension follow-up care and self-management, including cost, access to care, and transportation and wait time.

**Conclusion:**

Patients and physicians identified multiple barriers and facilitators to hypertension care. These perspectives may be helpful to design emergency department-based interventions that target blood pressure control and linkage to outpatient care.

## Introduction

Hypertension is a global epidemic with a growing burden in low- and middle-income countries (LMICs), specifically in sub-Saharan Africa (SSA) [1,2]. In northern Tanzania, the community prevalence of hypertension is 28–40% and is projected to increase substantially in the future [3–6]. Community and clinic-based screening health programs have been established to address this emerging epidemic; however, uptake is generally poor, success has been limited, and both undiagnosed and uncontrolled hypertension remain common [6–8].

Major drivers of the disparities in hypertension outcomes are lack of awareness and poor adherence to outpatient treatment [9,10]. Previously described barriers to successful outpatient care include limited health literacy [11], poor communication and structural barriers such as high cost of care and long transport time to clinic [5,11], and despite recent government investment in noncommunicable diseases, a 2018 study found that three fourths of outpatient facilities in Tanzania lack basic equipment such as blood pressure cuffs and are unprepared to manage hypertension screening and treatment [10].

Given these obstacles, understanding the targetable barriers and facilitators of hypertension care are crucial to developing novel strategies to provide optimal care. Given limited outpatient capacity for managing hypertension, patients with hypertension may seek care in hospital-based emergency departments (EDs). While not ideal, the ED has become a well-established source of healthcare for those with limited health care access and barriers to health maintenance. Indeed, recent studies from northern Tanzania found large numbers of patients with poorly controlled or undiagnosed hypertension presenting to the ED for care of their hypertension or hypertension-related complications [12,13]. Additionally, many patients with undiagnosed hypertension may present to the ED for unrelated complaints [12,13]. The emergency department may represent a unique opportunity to improve hypertension screening for undiagnosed patients in addition to education and linkage to outpatient care for high-risk patients. Limited studies in high-income countires have demonstrated that ED patients are receptive to screening, education, and assistance with follow-up care [14]. However, few rigorous studies have explored the barriers and facilitators of hypertension care in the emergency department populations in low- and middle-income countries such as Tanzania. Additionally, there have been no studies looking at interventions to capitalize on the ED location to improve hypertension care in Tanzania. Understanding the motivation behind seeking care in the ED and provider viewpoints are essential first steps in creating novel targeted interventions to improve hypertension awareness and control. We therefore conducted a qualitative study to determine unique barriers to hypertension management and control for hypertensive patients presenting to the ED in northern Tanzania.

## Methods

### Ethics

The study protocol was approved by the Kilimanjaro Christian Medical College (KCMC) Ethics Committee (EC#502), the National Institute for Medical Research in Tanzania, and the Duke University Institutional Review Board (#Pro00081220) in Durham, North Carolina. Informed written consent was obtained from all participants. Additional information regarding the ethical, cultural, and scientific considerations specific to inclusivity in global research is included in the Supporting Information (S1 Checklist).

### Setting

The study was conducted at Kilimanjaro Christian Medical College (KCMC) in Moshi, Tanzania. This hospital is a regional referral center and serves a population of over 15 million residents [15]. The Kilimanjaro region has a community hypertension prevalence of 28% [5]. The KCMC ED sees approximately 90–100 patients per day (KCMC Traumatic Brain Injury Registry, unpublished results, Staton 2017), and the prevalence of hypertension among the ED patient population is 34% [13].

### Focus group recruitment

Recruitment was conducted by trained research nurses in the ED. A prospective observational study was conducted to determine the prevalence of hypertension and follow-up rates in the ED and detailed sampling methods for this study have been previously reported [12]. Briefly, all adults (≥18) were screened and inclusion criteria included a single blood pressure level of ≥160/100 mm Hg or a two-time average of ≥140/90 mm Hg. Those meeting study criteria for hypertension were invited to participate in the study. Overall, in the parent study 78.6% of participants were aware of their hypertension diagnosis and 65.3% were currently on anti-hypertensives [12]. Exclusion criteria included inability to consent due to severity of illness or pregnancy. As this study was focused on long-term hypertension management, pregnant women were excluded due to transient alterations in blood pressure that may occur during pregnancy. A subset of participants enrolled in the observational study was recruited for the qualitative study by purposive sampling to include a diversity of ages, sexes, and education in addition to varied awareness and treatment of hypertension. The observational and qualitative studies were conducted simultaneously. COREQ guidelines were used for development of methods for this study (S1 Appendix).

### Qualitative data collection

#### Focus group discussions

To explore patient collective views of barriers to optimal hypertension care, we conducted three focus group discussions (FGDs) with 7–9 patients each who met study criteria for hypertension between November and December 2017. Sessions were not grouped by age or gender In order to maintain a diversity of viewpoints and spur discussion. All sessions were semi-structured, open-ended, and probing. Sessions lasted for approximately three hours. Moderators followed a semi-structured guide and asked open-ended questions to assess patients’ hypertension management, including knowledge, attitudes, and perceptions in addition to barriers and facilitators of hypertension management. The focus group guide was developed using an inductive approach to the framework method [16]. This approach was based on our previously-developed model which explored determinants of biomedical healthcare utilization among individuals in Kilimanjaro with noncommunicable diseases (NCDs), including hypertension and informed by the Health Belief Model [17,18]. Briefly, the Health Belief Model suggests that a person’s belief in both the threat of a disease and efficacy of health action determine the likelihood they will adopt a recommended behavior [17]. After data reduction, we performed open-coding of transcripts which incorporated key aspects of social cognitive behavioral theory [19].

The discussion guide was initially written in English and then translated to Swahili by an independent bilingual team. All sessions were moderated by a Tanzanian member of the research team with over five years of training and experience in qualitative methodology and translation. All sessions were audio-recorded, and one note-taker transcribed and independently translated each session. The moderator then reviewed the transcripts to ensure accuracy. Team meetings were held after each session, and again following translation to debrief and iteratively adjust the semi-structured guide.

#### In-depth interviews

ED physicians were recruited by ED director referral for in-depth interviews. An in-depth interview guide was developed using the framework method [16]. The interviewer asked open-ended questions to assess the physicians perspectives on barriers to hypertension management. In total, three in-depth interviews were performed in English by a physician researcher (SWG). Sample size for the physician in-depth interviews was largely determined by availability of emergency room physicians and resources to complete the study. A sample of three physicians was deemed practicable, given participant and resource availability, and was sufficient due to the exploratory nature of this research. The interviews were performed in a private, easily accessible location agreed upon by both researcher and physician and lasted approximately one hour. The physicians ranged from first-year interns to supervising directors of the emergency department. These interviews were also audio-recorded and transcribed.

### Qualitative data analysis

#### Focus group discussions and in-depth interviews

The qualitative coding, analytic memos, and corresponding matrices were stored and analyzed using NVivov.10.0 (QRS International Pty Ltd, Melbourne, Australia). We conducted a thematic analysis of the qualitative data by applying an inductive approach to the framework method [16]. After data reduction, we performed open-coding of all transcripts. We used a ‘cultural insider’ (emic) and a ‘cultural outsider’ (etic) to independently code the data. The cultural insider was a Tanzanian researcher living in Moshi (MP) and the cultural outsider was a researcher from the United States (SWG). Comparisons were made between each code set and areas of disagreement were discussed and resolved by revisiting the data and building consensus. This approach allowed us to explore concepts that otherwise may have been overlooked or misinterpreted by either researcher individually. The codes were grouped together into categories, and we further used a coding index to formulate connections and explore relationships.

## Results

### Characteristics

Between November and December 2017, we conducted three focus group discussions with 24 total participants. The age of participants ranged from 32–74 years (IQR 12.3) and the majority were female (n = 16; 67%) (**Table 1**). Most of the participants resided in a rural location (n = 19; 79%). Overall, 71% of participants had a previous diagnosis of hypertension and 63% were currently taking anti-hypertensives. The characteristics of the three ED physicians participating in in-depth interviews are summarized in **Table 2**. They ranged in age from 25–30 years. None of the physicians received formal hypertension training.

**Table 1:** Characteristics of the focus group participants.

	FG01	FG02	FG03
Study Participants	ED Patients	ED Patients	ED Patients
**Participants (N)**	7	9	8
**Gender**			
**Male**	1 (14%)	4 (44%)	3 (38%)
**Female**	6 (86%)	5 (56%)	5 (63%)
**Education**			
**None**	0	1 (11%)	1 (13%)
**Primary**	3 (43%)	4 (44%)	4 (50%)
**Secondary**	2 (29%)	2 (22%)	2 (25%)
**University**	2 (29%)	2 (22%)	1 (13%)
**Occupation**			
**Farmer/Wage Earner**	2 (29%)	4 (44%)	4 (50%)
**Small Business**	2 (29%)	1 (11%)	1 (13%)
**Professional**	3 (43%)	3 (33%)	2 (25%)
**Unemployed**	0	1 (11%)	1 (13%)
**Residence**			
**Urban**	2 (29%)	2 (22%)	1 (13%)
**Rural**	5 (72%)	7 (78%)	7 (87%)
**Previous HTN diagnosis**			
**Yes**	5 (72%)	5 (56%)	7 (87%)
**No**	2 (29%)	4 (44%)	1 (13%)
**Currently on HTN medications**			
**Yes**	4 (57%)	5 (56%)	6 (75%)
**No**	3 (43%)	4 (44%)	2 (25%)

ED, Emergency Department; HTN, hypertension.

**Table 2:** Characteristics of the in-depth interview participants.

	In-depth Interviews
Study Participants	ED Physicians
** **	
**Participants (N)**	3
**Gender**	
**Male**	2
**Female**	1
**Years of Clinical Practice**	
**One**	1
**Two**	1
**Five or More**	1
**Residence**	
**Urban**	3
**Rural**	0

**Fig 1:**
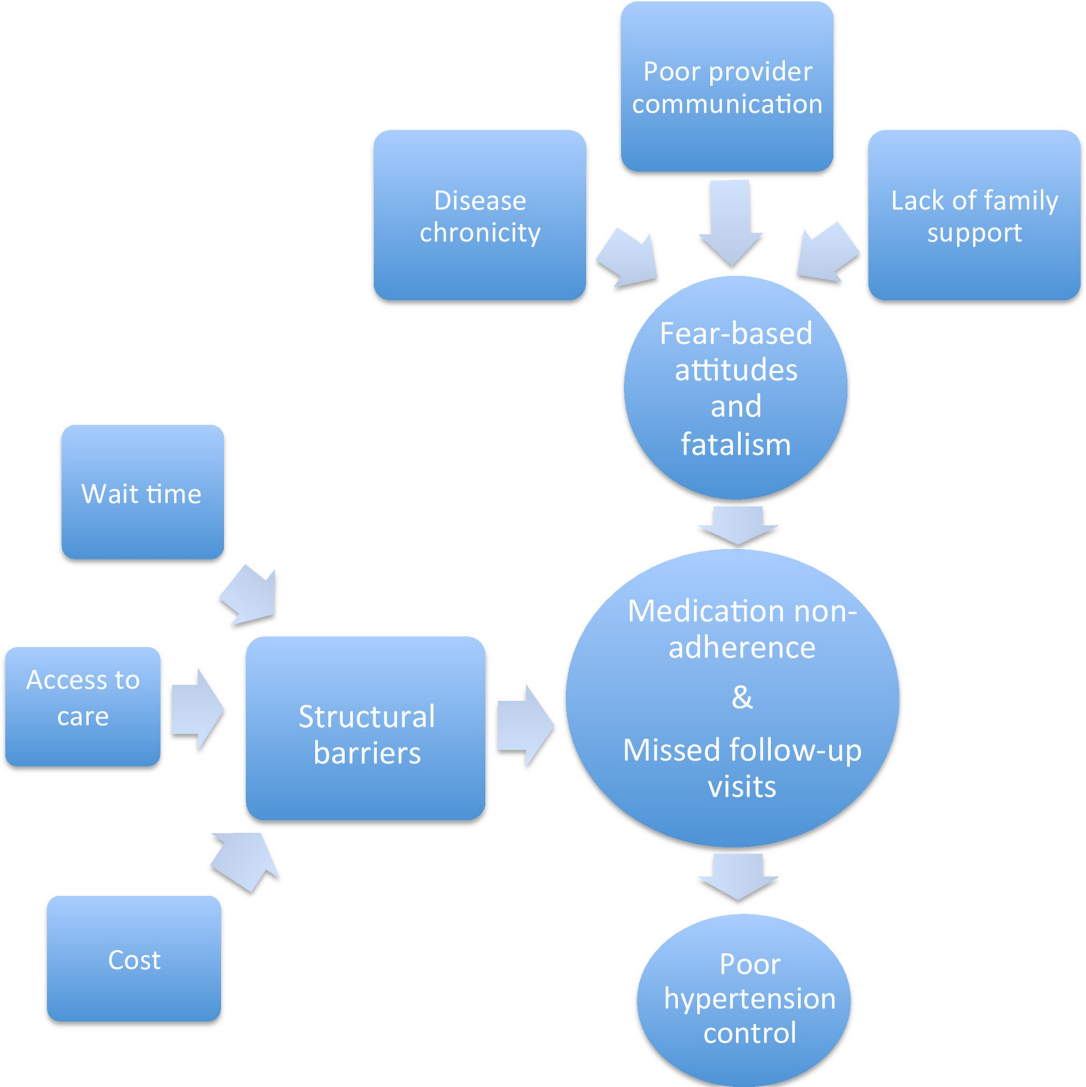
Model of barriers to hypertension care.

Overall, we identified two major domains: 1) patients’ knowledge, attitudes, and perceptions (KAP) towards their hypertension care and 2) structural barriers that limit hypertension control. Within the KAP domain, four major themes emerged including disease chronicity, provider communication, family support, and fear-based attitudes. Within the structural domain we identified several barriers that impeded optimal follow-up care and disease self-management, including cost, access to care, and transportation/wait time **(Fig 1).**

### Knowledge, attitudes, and perceptions towards hypertension care

#### Disease chronicity

Participants did not understand hypertension as a chronic condition. Some thought it could be cured permanently with either a short duration of biomedical medications or traditional medicines.

*This problem can be temporary and if you rest or get some traditional medicine then the problem subsides*. *(patient*, *age 45*, *female)*

Providers also agreed that the chronic nature of hypertension and the necessity of lifelong medications was not well understood among both patients and fellow providers.

*Even some doctors don’t understand that hypertension is chronic; they prescribe medicines for two weeks only and then tell patients to stop*. *(physician*, *age 29*, *female*)

*Maybe the doctors are not sure of the diagnosis; they are scared to tell the patients that it is for a lifetime*. *(physician*, *age 29*, *female*)

#### Provider communication

Participants described positive communication, especially two-way, open-ended communication, as an important factor in understanding hypertension and framing the disease from an optimistic manner. Frequent and positive communication encouraged patients to continue with the treatment course.

*I am always happy because the doctor asks me many questions and sometimes I laugh*, *so he makes me not forget medication or coming to clinic*, *and sometimes he calls and reminds me to take medications*. *(patient*, *age 51*, *female*)

*For sure*, *when I see a doctor I feel comforted because he asks me a lot of questions and he tests my pressure; when he found that the pressure is high*, *he asks me politely*, *“What troubles you this week*?*” And I explain to him and he gives me the answer and advice*. *Then I feel so good*! *(patient*, *age 74*, *female*)

Poor communication was also a common concern and many participants described inadequate explanations from the provider leading to fear and misunderstanding regarding hypertension.

*In the emergency room there are some young doctors who don’t want to listen to us*. *They are busy with phones even while talking to them*, *and if you say that these drugs you prescribed to me last visit have a lot of side effects*, *they answer us rudely*. *They don’t respect us […] and when we go back home our pressure is higher than before we went to hospital*! *(patient*, *age 55*, *female*)

*This doctor makes my pressure go high*. *From what I know*, *a good doctor must be patient and a good listener*. *He was talking to me like a policeman*. *I felt really bad*. *(patient*, *age 63*, *female*)

#### Family support

Conversely, participants described family support as an important factor in adhering to their hypertension care.

*And if your child or wife knows your status it will be easy for them to remind you that at a certain time you have to take medication; if your time for medication is morning*, *or evening*, *they will remind you*. *So it is important that the person you’re living with knows everything about your health (patient*, *age 54*, *female*).

#### Fear-based attitudes

The chronic nature of hypertension and poor communication lead some participants to have fear surrounding the idea of hypertension. This often lead to misunderstanding, hopelessness, and fatalism.

*When someone tells a young person that they have high blood pressure it is difficult to receive*. *(patient*, *age 54*, *female*)

*I was scared*! *My heart was running [*…*] then I understood and become worried and I started using medications*. *(patient*, *age 76*, *female*)

*I’ve been trying not to think more about my disease*, *because when you think more you end up with losing hope and the pressure starts to elevate again*. *(patient*, *age 32*, *male*)

*They [physicians] will create a idea that the patient’s whole family and generation will die with hypertension*. *(patient*, *age 32*, *male*)

### Structural barriers that limit hypertension control

Participants and providers also reported multiple intersecting structural barriers including transportation/wait time at clinics, access to care, and cost which directly impacted their hypertension control.

#### Wait time/transportation time

Patients reported having to wait for transportation to bring them to and from the clinic. In addition, they described substantially long wait times at the clinic itself, even when they had an appointment to see the physician at a specific time. These long wait times may have led to disengagement and nonadherence to their hypertension care.

*If I come to hospital I must follow the queue so this costs me a lot of time; that is why I wait two to three days without medication and my condition becomes worse and I get seriously sick*. *(patient*, *age 63*, *male*)

#### Access to care

Patients in rural areas described difficulty in obtaining high-quality care. Physicians also confirmed that access to care was a challenge in remote areas.

*Many doctors are not willing to go into rural areas so the patients in the rural areas suffer*. *The quality of the service provided is not the same as in town*. *Some of them are poorly managed*. *(physician*, *age 30*, *male*)

#### Cost

Transportation cost was cited as a major barrier to care for many participants. However, medication cost and availability was rarely mentioned as a barrier. This may be unique to this region and the KCMC population due to readily available access of a tertiary care hospital. As one doctor described:

*Many medicines are common [*…*] nifedipine*, *atenolol*, *Lasix are available everywhere*. *(physician*, *age 28*, *female*)

## Discussion

Our study explored ED patient and provider perspectives regarding hypertension management in northern Tanzania. We found that poor communication between providers and patients and lack of disease understanding contributed to fear-based attitudes regarding hypertension diagnosis. Conversely, in this sample, positive communication and family support played an outsized role in facilitating follow-up care and individual agency. As in previous studies in SSA, structural barriers also impeded care continuity and necessary follow-up for medication titration. Multifacteted, novel ED-based interventions that focus on provider and patient knowledge acquisition and communication while engaging family members in care should be considered in the future.

Patients and some providers had difficulty understanding the chronic nature of hypertension and most were unaware that the condition required lifelong medication. This was not surprising as similar results from qualitative studies have been seen in this region and in other areas throughout SSA [12,20–22]. For example, a study in Nigeria found that the majority of participants with hypertension believed it was a temporary condition [23]. Poor understanding of disease chronicity was compounded by reluctance or inability of physicians to communicate the expected disease course and lifelong treatment plan. Based on our results, communication barriers included inadequate time for counseling and limited provider knowledge. As this study was conducted in an ED population with patients essentially receiving primary care services from an ED provider, lack of time and focus to create a therapeutic relationship was a major factor in communication challenges. Previous studies have also shown that time constraints lead to less guideline adherence [24] and more unecessary prescriptions [25]. Additionally, many ED providers are new physicians with limited experience and oversight and may lack the skills to properly explain this disease course or the urgency and importance of follow-up care [26]. The combination of limited time and limited experience likely results in overall poor patient knowledge and disease comprehension.

We found that the chronic nature of hypertension, lack of disease understanding and poor provider support contributed to fear surrounding hypertension. Providers were reluctant to alarm or “frighten” the patient by emphasizing the lifelong course of hypertension. A recent study in Kenya also described fear as a major barrier to hypertension communication and care [27] and a study in the Kilimanjaro region found that if patients expressed fear of their hypertension diagnosis then they were significantly less likely to present for follow-up [12]. It is less clear exactly how fear contributes to a lack of follow-up and linkage to care. One explanation is that this fear leads some patients to develop fatalistic attitudes and subsequently a lack of perceived individual agency in controlling their hypertension [19]. Fatalism, the idea that that fate governs major life outcomes, is a cultural belief that has been well described throughout SSA and seems to be a pervasive attitude in the Kilimanjaro region [20,28–30]. Previous studies throughout SSA have also noted a fatalistic attitude towards other chronic diseases such as HIV [31] and one study found that fatalism increases in response to a new chronic disease diagnosis [32]. Additionally, in line with social cognitive theory, the degree of fatalism and self-efficacy may also be predictive of chronic disease management and outcomes [33,34]. Further focused research is needed to better understand how fatalistic attitudes are developed and what protective factors might foster resilience and individual agency in chronic disease management.

We found that knowledge, communication, and family support are important to patients; thus improving patient and provider knowledge and communication may be highly impactful and may serve to improve individual agency, mitigate fatalistic attitudes and behaviors, and potentially improve hypertension control [35]. Therefore, future interventions should focus on improving the provider/patient relationship and communication [36]. Given physician time and resource demands in the ED, this type of intervention could be conducted by a nurse or mid-level provider [37,38]. In fact, a recent study of a hypertension intervention in the hypertensive HIV population in the Kilimanjaro region found a brief educational intervention delivered by a community health worker in the outpatient setting to be well accepted, highly feasible and potentially efficacious [39]. Two large-scale randomized, controlled trials incorporating community health workers for hypertension in Kenya found similar positive results [40,41]. We propose a similar intervention, utilizing a community health worker or nurse to deliver hypertension counseling and education, which could be helpful in the ED population as well. We recommend development of an evidence-based targeted educational intervention for ED patients focused on improving individual agency and self-efficacy [35,42,43]. In addition to focusing on tempering fatalistic attitudes and providing support, this future intervention should engage family members by offering education and linking them into the patient’s treatment plan. This novel educational intervention may use visual aides and/or mobile technology [44] as well to aid knowledge transfer and must be developed iteratively with key stakeholders, patients, and providers. Further research is necessary to develop this intervention and test the effectiveness at improving blood pressure control and follow-up in Northern Tanzania. If successful, this intervention could be scaled-up to similar EDs throughout SSA.

In addition to education, structural barriers must also be addressed. The majority of structural barriers to care found in this study were rooted to physical distance to high quality outpatient healthcare. Frustrations with transportation time, transportation cost and accessibility of care were emphasized by most participants. While resources have recently been allocated towards hypertension screening with modest improvements in diagnosis rates [10], patients are often lost to follow-up and cease medical treatment due to frustrations with access and travel to appointments [12]. This is detrimental as blood pressure control often requires two or more medications titrated over a number of outpatient visits [45,46]. Unfortunately, the majority of rural outpatient clinics in Tanzania lack basic equipment and are unprepared to manage hypertension treatment [10] and thus many patients travel long distances and seek care in the tertiary care settings like the KCMC ED. In fact, a recent study by our group found that 32.6% of all KCMC ED visits were for routine hypertension care and medication refills [12]. It is concerning that Tanzania has surpassed the United States in this regard where approximately one fourth of ED visits are for hypertension primary care [47]. In order to reverse this trend, we must first fully accept that the ED has become a well-established source of healthcare and shift resources accordingly. In order for the ED to adopt this public health mission, more dedicated personnel are needed to provide hypertension educational interventions and promote linkage to appropriate venues for longitudinal medication titration. Simultaneous expansion of remote hypertension clinics and outreach programs that bring care directly to patients are needed as well [48]. Enhanced referral to these outpatient facilities with possible incentivization must also be facilitated in the ED. Specifically, we suggest direct scheduling of follow-up appointments from the ED and possible mobile transportation vouchers [49] to facilitate follow-up care as these techniques have been successful in similar settings [50–52]. While these interventions do require an initial investment of substantial resources, we anticipate that they will save lives and money due to long-term reduction of hypertension-related complications and death.

### Limitations

This study had several limitations. First, this sample was from a tertiary referral hospital, so patients may have higher access to care than the general population thus limiting generalizability. Social desirability bias was likely present during the physician interviews as these were conducted by one of the non-Tanzanian investigators. In addition, the structured interviews and inductive approach could limit possible new emerging themes. In contrast, the focus groups were less structured and more open-ended, allowing for the emergence of new themes. The number of interviews and focus groups were also limited by budget constraints; a larger, more diverse sample would ensure a more representative range of ideas and would be more informative. Finally, the same investigator who conducted the interviews also performed coding and developed the codebook and this may have introduced bias; however, this was mitigated by using a cultural insider as the secondary coder.

## Conclusions

Sub-Saharan Africa has one of the highest prevalences of hypertension in the world. As cardiovascular disease and hypertension make up an increasingly large proportion of the public health agenda in Tanzania, the ED should be prioritized as an important site for screening and linkage to longitudinal care. Development of a targeted ED- based educational intervention is needed to address both individual agency and care continuity. Policy-makers should focus on ensuring availablity of resources needed for aggressive expansion and prioritization of care in the remote regions coupled with ED-based referrals including direct scheduling and incentivization. Multi-faceted interventions are urgently needed to improve hypertension control and prevent future cardiovascular death.

## Supporting information

S1 Checklist Inclusivity in global research checklist. (DOCX)

S1 Appendix COREQ guidelines. (DOCX)

## References

[1] Nahimana MR, Nyandwi A, Muhimpundu MA, Olu O, Condo JU, Rusanganwa A, et al. A population-based national estimate of the prevalence and risk factors associated with hypertension in Rwanda: implications for prevention and control. BMC Public Health. 2017;18(1):2. Epub 2017/07/12. doi: 10.1186/s12889-017-4536-9 ; PubMed Central PMCID: PMC5504833. 28693458 PMC5504833

[2] Price AJ, Crampin AC, Amberbir A, Kayuni-Chihana N, Musicha C, Tafatatha T, et al. Prevalence of obesity, hypertension, and diabetes, and cascade of care in sub-Saharan Africa: a cross-sectional, population-based study in rural and urban Malawi. The lancet Diabetes & endocrinology. 2018;6(3):208–22. Epub 2018/01/27. doi: 10.1016/S2213-8587(17)30432-1 ; PubMed Central PMCID: PMC5835666. 29371076 PMC5835666

[3] Khamis AG, Senkoro M, Mwanri AW, Kreppel K, Mfinanga SG, Bonfoh B, et al. Prevalence and determinants of hypertension among pastoralists in Monduli District, Arusha region in Tanzania: a cross-sectional study. Archives of public health = Archives belges de sante publique. 2020;78:99. Epub 2020/10/20. doi: 10.1186/s13690-020-00485-0 ; PubMed Central PMCID: PMC7556965. 33072318 PMC7556965

[4] Mathers CD, Loncar D. Projections of Global Mortality and Burden of Disease from 2002 to 2030. PLOS Medicine. 2006;3(11):e442. doi: 10.1371/journal.pmed.0030442 17132052 PMC1664601

[5] Galson SW, Staton CA, Karia F, Kilonzo K, Lunyera J, Patel UD, et al. Epidemiology of hypertension in Northern Tanzania: a community-based mixed-methods study. BMJ open. 2017;7(11):e018829. Epub 2017/11/12. doi: 10.1136/bmjopen-2017-018829 ; PubMed Central PMCID: PMC5695455. 29127232 PMC5695455

[6] Muhamedhussein MS, Nagri ZI, Manji KP. Prevalence, Risk Factors, Awareness, and Treatment and Control of Hypertension in Mafia Island, Tanzania. International journal of hypertension. 2016;2016:1281384. doi: 10.1155/2016/1281384 ; PubMed Central PMCID: PMC4971322. 27525113 PMC4971322

[7] Manavalan P, Madut DB, Hertz JT, Thielman NM, Okeke NL, Mmbaga BT, et al. Hypertension burden and challenges across the hypertension treatment cascade among adults enrolled in HIV care in northern Tanzania. Journal of clinical hypertension (Greenwich, Conn). 2020;22(8):1518–22. Epub 2020/07/12. doi: 10.1111/jch.13929 ; PubMed Central PMCID: PMC7719079. 32652868 PMC7719079

[8] Paccaud PB, Jean-Pierre G, Mashombo M, Marianna B, Christian L, Fred. Low utilization of health care services following screening for hypertension in Dar es Salaam (Tanzania): a prospective population-based study. BMC Public Health. 2008;8(1):1. doi: 10.1186/1471-2458-8-407 19087300 PMC2615777

[9] Addo J, Smeeth L, Leon DA. Hypertension in sub-saharan Africa: a systematic review. Hypertension. 2007;50(6):1012–8. Epub 10/24. doi: 10.1161/HYPERTENSIONAHA.107.093336 . 17954720

[10] Bintabara D, Mpondo BCT. Preparedness of lower-level health facilities and the associated factors for the outpatient primary care of hypertension: Evidence from Tanzanian national survey. PloS one. 2018;13(2):e0192942. Epub 2018/02/16. doi: 10.1371/journal.pone.0192942 ; PubMed Central PMCID: PMC5814020. 29447231 PMC5814020

[11] Naanyu V, Vedanthan R, Kamano JH, Rotich JK, Lagat KK, Kiptoo P, et al. Barriers Influencing Linkage to Hypertension Care in Kenya: Qualitative Analysis from the LARK Hypertension Study. Journal of general internal medicine. 2016;31(3):304–14. Epub 01/04. doi: 10.1007/s11606-015-3566-1 . 26728782 PMC4762819

[12] Galson SW, Stanifer JW, Hertz JT, Temu G, Thielman N, Gafaar T, et al. The burden of hypertension in the emergency department and linkage to care: A prospective cohort study in Tanzania. PloS one. 2019;14(1):e0211287. Epub 2019/01/27. doi: 10.1371/journal.pone.0211287 ; PubMed Central PMCID: PMC6347227. 30682173 PMC6347227

[13] Hertz JT, Sakita FM, Manavalan P, Madut DB, Thielman NM, Mmbaga BT, et al. The Burden of Hypertension and Diabetes in an Emergency Department in Northern Tanzania. Ethnicity & disease. 2019;29(4):559–66. doi: 10.18865/ed.29.4.559 ; PubMed Central PMCID: PMC6802168. 31641323 PMC6802168

[14] Shah T, Aronow WS, Peterson SJ, Goldwag D. Diagnosis, treatment, and referral of hypertension or prehypertension in an emergency department after an educational program: preliminary results. Journal of clinical hypertension (Greenwich, Conn). 2011;13(6):413–5. Epub 2011/06/09. doi: 10.1111/j.1751-7176.2010.00423.x ; PubMed Central PMCID: PMC8108879. 21649840 PMC8108879

[15] The Community Health Fund Act. Tanzania2001.

[16] Gale NK, Heath G, Cameron E, Rashid S, Redwood S. Using the framework method for the analysis of qualitative data in multi-disciplinary health research. BMC medical research methodology. 2013;13:117. Epub 09/21. doi: 10.1186/1471-2288-13-117 . 24047204 PMC3848812

[17] Rosenstock IM, Strecher VJ, Becker MH. Social Learning Theory and the Health Belief Model. Health Education Quarterly. 1988;15(2):175–83. doi: 10.1177/109019818801500203 3378902

[18] Stanifer JW, Patel UD, Karia F, Thielman N, Maro V, Shimbi D, et al. The determinants of traditional medicine use in Northern Tanzania: a mixed-methods study. PloS one. 2015;10(4):e0122638. Epub 2015/04/08. doi: 10.1371/journal.pone.0122638 ; PubMed Central PMCID: PMC4388565. 25848762 PMC4388565

[19] Bandura A. Human agency in social cognitive theory. American psychologist. 1989;44(9):1175. doi: 10.1037/0003-066x.44.9.1175 2782727

[20] Manavalan P, Minja L, Wanda L, Hertz JT, Thielman NM, Okeke NL, et al. "It’s because I think too much": Perspectives and experiences of adults with hypertension engaged in HIV care in northern Tanzania. PloS one. 2020;15(12):e0243059. Epub 2020/12/04. doi: 10.1371/journal.pone.0243059 ; PubMed Central PMCID: PMC7714125. 33270765 PMC7714125

[21] Nyaaba GN, Masana L, Aikins Ad-G, Stronks K, Agyemang C. Lay community perceptions and treatment options for hypertension in rural northern Ghana: a qualitative analysis. BMJ open. 2018;8(11). doi: 10.1136/bmjopen-2018-023451 30498042 PMC6278795

[22] Murphy K, Chuma T, Mathews C, Steyn K, Levitt N. A qualitative study of the experiences of care and motivation for effective self-management among diabetic and hypertensive patients attending public sector primary health care services in South Africa. BMC health services research. 2015;15(1):303. doi: 10.1186/s12913-015-0969-y 26231178 PMC4522057

[23] Osamor PE, Owumi BE. Factors associated with treatment compliance in hypertension in southwest Nigeria. Journal of health, population, and nutrition. 2011;29(6):619. doi: 10.3329/jhpn.v29i6.9899 22283036 PMC3259725

[24] Tsiga E, Panagopoulou E, Sevdalis N, Montgomery A, Benos A. The influence of time pressure on adherence to guidelines in primary care: an experimental study. BMJ open. 2013;3(4). doi: 10.1136/bmjopen-2013-002700 23585394 PMC3641486

[25] Dugdale DC, Epstein R, Pantilat SZ. Time and the patient–physician relationship. Journal of general internal medicine. 1999;14(Suppl 1):S34. doi: 10.1046/j.1525-1497.1999.00263.x 9933493 PMC1496869

[26] Edward A, Hoffmann L, Manase F, Matsushita K, Pariyo GW, Brady TM, et al. An exploratory study on the quality of patient screening and counseling for hypertension management in Tanzania. PloS one. 2020;15(1):e0227439. Epub 2020/01/17. doi: 10.1371/journal.pone.0227439 ; PubMed Central PMCID: PMC6964881 Johns Hopkins. FM was the director of CCPmedicine. This does not alter our adherence to PLOS ONE policies on sharing data and materials. We have no other competing interests to disclose. 31945075 PMC6964881

[27] Naanyu V, Vedanthan R, Kamano JH, Rotich JK, Lagat KK, Kiptoo P, et al. Barriers influencing linkage to hypertension care in Kenya: qualitative analysis from the LARK hypertension study. Journal of general internal medicine. 2016;31(3):304–14. doi: 10.1007/s11606-015-3566-1 26728782 PMC4762819

[28] Iliffe J. Africans: The history of a continent: Cambridge University Press; 2017.

[29] Gannon MJ, Pillai R. Understanding global cultures: Metaphorical journeys through 34 nations, clusters of nations, continents, and diversity: Sage Publications; 2015.

[30] Hicks D. M. Fortes, Oedipus and Job in West African Religion. With an essay by Robin Horton. Homme. 1984;24(3):139–40.

[31] Caldwell JC. Rethinking the African AIDS epidemic. Population and development review. 2000;26(1):117–35.

[32] Meyer-Weitz A. Understanding fatalism in HIV/AIDS protection: the individual in dialogue with contextual factors. African Journal of AIDS Research. 2005;4(2):75–82. doi: 10.2989/16085900509490345 25870883

[33] Franklin MD, Schlundt DG, McClellan LH, Kinebrew T, Sheats J, Belue R, et al. Religious fatalism and its association with health behaviors and outcomes. American journal of health behavior. 2007;31(6):563–72. doi: 10.5555/ajhb.2007.31.6.563 17691869 PMC4144788

[34] Bandura A. Self-efficacy: Toward a unifying theory of behavioral change. Psychological Review. 1977;84:191–215. doi: 10.1037//0033-295x.84.2.191 847061

[35] Koffi J, Konin C, Gnaba A, NGoran Y, Mottoh N, Guikahue M, editors. Usefulness of patient education in antihypertensive treatment compliance in black Africans. Annales de Cardiologie et D’angeiologie; 2017. 10.1016/j.ancard.2017.04.012 28935205

[36] Goverwa TP, Masuka N, Tshimanga M, Gombe NT, Takundwa L, Bangure D, et al. Uncontrolled hypertension among hypertensive patients on treatment in Lupane District, Zimbabwe, 2012. BMC research notes. 2014;7(1):703. doi: 10.1186/1756-0500-7-703 25297796 PMC4197296

[37] Spies LA, Bader SG, Opollo JG, Gray J. Nurse-Led Interventions for Hypertension: A Scoping Review With Implications for Evidence-Based Practice. Worldviews on evidence-based nursing. 2018;15(4):247–56. Epub 2018/06/15. doi: 10.1111/wvn.12297 . 29902358

[38] Gyamfi J, Plange-Rhule J, Iwelunmor J, Lee D, Blackstone SR, Mitchell A, et al. Training nurses in task-shifting strategies for the management and control of hypertension in Ghana: a mixed-methods study. BMC health services research. 2017;17(1):1–9. 28148255 10.1186/s12913-017-2026-5 PMC5288999

[39] Manavalan P, Madut DB, Wanda L, Msasu A, Mmbaga BT, Thielman NM, et al. A community health worker delivered intervention to address hypertension among adults engaged in HIV care in northern Tanzania: Outcomes from a pilot feasibility study. The Journal of Clinical Hypertension. 2022;24(8):1095–104. doi: 10.1111/jch.14518 35899325 PMC9380132

[40] Ogola EN, Okello FO, Herr JL, Macgregor-Skinner E, Mulvaney A, Yonga G. Healthy Heart Africa–Kenya: A 12-Month Prospective Evaluation of Program Impact on Health Care Providers’ Knowledge and Treatment of Hypertension. Global heart. 2019;14(1):61–70. doi: 10.1016/j.gheart.2019.02.002 31036303

[41] Yonga G, Okello FO, Herr JL, Mulvaney A, Ogola EN. Healthy Heart Africa: a prospective evaluation of programme outcomes on individuals’ hypertension awareness, screening, diagnosis and treatment in rural Kenya at 12 months. Cardiovasc J Afr. 2020;31(1):9–15. Epub 2019/11/30. doi: 10.5830/CVJA-2019-037 . 31781714 PMC8762776

[42] Boulware LE, Daumit GL, Frick KD, Minkovitz CS, Lawrence RS, Powe NR. An evidence-based review of patient-centered behavioral interventions for hypertension11The full text of this review article is available via AJPM Online at http://www.elsevier.com/locate/ajpmonline. American Journal of Preventive Medicine. 2001;21(3):221–32. doi: 10.1016/S0749-3797(01)00356-7 11567845

[43] Gleason-Comstock J, Streater A, Ager J, Goodman A, Brody A, Kivell L, et al. Patient education and follow-up as an intervention for hypertensive patients discharged from an emergency department: a randomized control trial study protocol. BMC Emergency Medicine. 2015;15(1):38. doi: 10.1186/s12873-015-0052-3 26691646 PMC4687379

[44] Bloomfield GS, Vedanthan R, Vasudevan L, Kithei A, Were M, Velazquez EJ. Mobile health for non-communicable diseases in Sub-Saharan Africa: a systematic review of the literature and strategic framework for research. Globalization and health. 2014;10:49. Epub 06/15. doi: 10.1186/1744-8603-10-49 . 24927745 PMC4064106

[45] Carey RM, Whelton PK. Prevention, detection, evaluation, and management of high blood pressure in adults: synopsis of the 2017 American College of Cardiology/American Heart Association Hypertension Guideline. Annals of internal medicine. 2018;168(5):351–8. doi: 10.7326/M17-3203 29357392

[46] Williams B, Mancia G, Spiering W, Agabiti Rosei E, Azizi M, Burnier M, et al. 2018 ESC/ESH Guidelines for the management of arterial hypertension: The Task Force for the management of arterial hypertension of the European Society of Cardiology (ESC) and the European Society of Hypertension (ESH). European heart journal. 2018;39(33):3021–104. 30165516 10.1093/eurheartj/ehy339

[47] McNaughton CD, Self WH, Zhu Y, Janke AT, Storrow AB, Levy P. Incidence of Hypertension-Related Emergency Department Visits in the United States, 2006 to 2012. The American journal of cardiology. 2015;116(11):1717–23. Epub 10/12. doi: 10.1016/j.amjcard.2015.09.007 Epub 2015 Sep 10. . 26454813 PMC4648677

[48] van den Berg MJ, van Loenen T, Westert GP. Accessible and continuous primary care may help reduce rates of emergency department use. An international survey in 34 countries. Family Practice. 2015;33(1):42–50. doi: 10.1093/fampra/cmv082 26511726

[49] Nzorubara D, Ommeh M, Fenenga C, Hesp C, Rinke de Wit T. Using mobile transport vouchers to improve access to skilled delivery. Rural and Remote Health. 2019;19:1–6. doi: 10.22605/RRH4577 30736701

[50] Atzema CL, Maclagan LC. The Transition of Care Between Emergency Department and Primary Care: A Scoping Study. Academic Emergency Medicine. 2017;24(2):201–15. doi: 10.1111/acem.13125 27797435

[51] Messina FC, McDaniel MA, Trammel AC, Ervin DR, Kozak MA, Weaver CS. Improving specialty care follow-up after an ED visit using a unique referral system. Am J Emerg Med. 2013;31(10):1495–500. Epub 2013/09/17. doi: 10.1016/j.ajem.2013.08.007 . 24035046

[52] Smith SR, Jaffe DM, Fisher EB Jr., Trinkaus KM, Highstein G, Strunk RC. Improving follow-up for children with asthma after an acute Emergency Department visit. The Journal of pediatrics. 2004;145(6):772–7. Epub 2004/12/08. doi: 10.1016/j.jpeds.2004.08.029 . 15580199

